# Effects of urbanization on the foraging ecology and microbiota of the generalist seabird *Larus argentatus*

**DOI:** 10.1371/journal.pone.0209200

**Published:** 2018-12-18

**Authors:** Matthew Fuirst, Richard R. Veit, Megan Hahn, Nolwenn Dheilly, Lesley H. Thorne

**Affiliations:** 1 School of Marine and Atmospheric Sciences, Stony Brook University, Stony Brook, NY, United States of America; 2 Department of Biology, College of Staten Island (CSI) CUNY, Staten Island, NY, United States of America; University of Illinois at Urbana-Champaign, UNITED STATES

## Abstract

*Larus* gull species have proven adaptable to urbanization and due to their generalist feeding behaviors, they provide useful opportunities to study how urban environments impact foraging behavior and host-associated microbiota. We evaluated how urbanization influenced the foraging behavior and microbiome characteristics of breeding herring gulls (*Larus argentatus*) at three different colonies on the east coast of the United States. Study colonies represented high, medium and low degrees of urbanization, respectively. At all colonies, gulls frequently foraged at landfills and in other urban environments, but both the use of urban environments and gull foraging metrics differed with the degree of urbanization. Gulls at the more urban colonies used urban environments more frequently, showed higher rates of site fidelity and took shorter trips. Gulls at less urban colonies used a greater diversity of habitat types and foraged offshore. We observed high microbial diversity at all colonies, though microbial diversity was highest at the least urban colony where gulls used a wider variety of foraging habitats. This suggests that gulls may acquire a wider range of bacteria when visiting a higher variety of foraging sites. Our findings highlight the influence of urban habitats on gull movements and microbiome composition and diversity during the breeding season and represent the first application of amplicon sequence variants, an objective and repeatable method of bacterial classification, to study the microbiota of a seabird species.

## Introduction

Cities are rapidly expanding, and the consequent urbanization of natural landscapes has widespread effects on wildlife ecology, biodiversity and ecosystem structure and function [1–3]. For animals living in urban environments, interactions with urban landscapes can influence animal movement, foraging behavior, predation risk, and reproductive success [4–11]. Despite the rapid increase of urbanization and its subsequent impact on wildlife ecology at a global scale [12], ecologists typically focus research on animals in natural environments, whereas studies of animal behavior within urban environments are more limited [12–14]. Urban ecosystems, where humans and wild animals coexist, are important regions to study when considering microbial transfer between organisms [6,15,16]. Targeted studies along urban gradients could greatly improve our understanding of how urban pressure influences animal behavior and animal health.

The foraging activities and habitat use of wild animals can be altered by urbanization. For example, the density and accessibility of food sources due to modification of habitats can alter the dietary and spatial patterns of animals [17–19]. Species with high plasticity in their foraging behavior can take advantage of these novel foraging areas, which allows them to occupy a wide array of habitats [8,20,21]. However, contact with urban environments can also affect exposure to pollutants and human-associated bacteria and viruses [5–7,10,11].

Microbes play a key role in physiology, influencing nutritional, immunological, physiological, and behavioral processes [22–24]. The holobiont is defined as the combination of the host and all of its associated microbes [25]. In particular, the microbiome, which refers to the genomic information carried by an entire community of microorganisms (including bacteria, archaea, fungi, protists, and viruses) of a certain body site or environment [26,27], is a vital indicator of animal health. The microbiota of wild animals can be influenced by exposure to microorganisms through variation in diet or habitat [28–30]. For example, urban habitats can cause changes to the gut microbiota of wild birds [31,32] and can act as sources of pathogens and contaminants [16,33]. Animal movement provides a powerful means of assessing the impacts of urbanization on wildlife and, when paired with microbial studies, can be valuable for the management of highly mobile species that may serve as vectors of microbes as they move across large spatial areas and interact with other animals. Migratory behaviors can increase the geographic spread of diseases [16,34–36] and pathogen exchange can be enhanced at feeding, breeding, and roosting grounds, where animals spend long periods of time in close physical contact with one another [37–39]. Studies investigating how foraging behavior and the use of human-altered habitats influence the microbiota of wild animals are needed to improve our understanding of the transfer of microbiota between humans and wildlife, particularly in urban landscapes where humans and wild animals frequently interact [15,40,41].

Gulls are ideal study species to investigate the effects of urbanization on microbial communities; they thrive in urban environments, are highly mobile, make use of a variety of natural and urbanized habitats, and are reservoirs for pathogens [4,8,17,21,42–47]. In addition, when gulls are breeding, they can easily be captured to acquire microbial samples [48]. Avian microbial communities are known to be influenced by physical location, phylogeny, and breeding ecology, but the effect of foraging behavior and habitat use on microbial composition and diversity is often overlooked [49–52]. Several studies on gulls and locations that gulls frequent have identified the presence of bacteria such as *Escherichia coli*, *Campylobacter sp*., *Clostridium sp*., and *Salmonella*, which typically colonize the gut of some organisms with no harmful effects, but are also known to cause disease in vertebrate animals [53–59]. However, little is known about how gull foraging movements, habitat use, or colony location can influence the composition and diversity of their microbiota.

Here we address this gap in knowledge by integrating movement and microbial studies of herring gulls from three breeding colonies on the east coast of the United States representing high, medium and low degrees of urbanization, respectively, to examine how urbanization influences the foraging behavior and microbiome of gulls.

## Methods

### Study species and sample collection

Consistent with gulls worldwide, herring gulls underwent dramatic population growth and expansion during the late 19^th^ century, which continued through the late 20^th^ century [4,44]. Herring gulls expanded their range from northern New England and Canadian Maritime provinces south to the mid-Atlantic Coast leading to populations reaching the tens of thousands [4]. Their current foraging behavior and habitat use need to be interpreted within the context of these dramatic changes in distribution and abundance, which were precipitated mainly by increasing urbanization.

**Fig 1:**
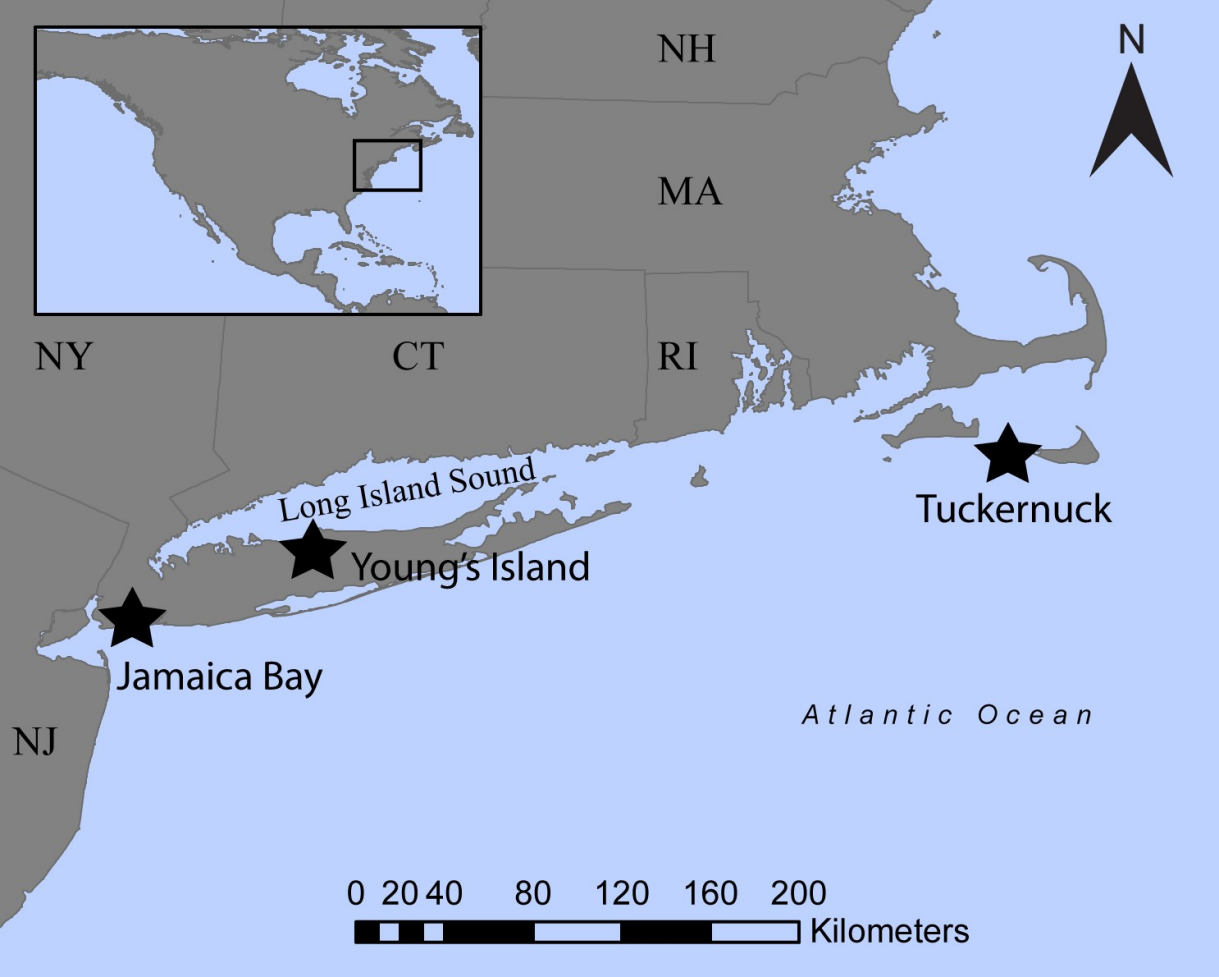
Locations of the study sites at three breeding colonies of herring gulls *Larus argentatus*. U.S. states included in the map of the Northeast U.S. are New Jersey (NJ), New York (NY), Connecticut (CT), Rhode Island (RI), Massachusetts (MA), and New Hampshire (NH).

We studied herring gulls at three islands along the east coast of the U.S. from late April to late June of 2016–2017 when gulls were incubating eggs (Fig 1). The Jamaica Bay Wildlife Refuge (JB) (40.59° N, -73.83° W) within New York City (NYC) was the most urbanized location (population density of 27,000 people per square mile within NYC) [60], while Young’s Island (YI) (40.92° N, -73.15° W) in Suffolk County on Long Island, New York was intermediate (population density of Suffolk Count of 1,639 people per square mile) [61], and Tuckernuck Island (TN) (41.29° N, -70.24° W) west of Nantucket, Massachusetts was the least urban environment (population density of 10 people per square mile) [62]. Commercial fishing vessels, which primarily fish for squid and bluefish, frequently use the waters around Tuckernuck, MA in spring.

We assessed the foraging movements of 45 gulls (JB = 15, YI = 15, TN = 15) during the incubation period using igotU GT-120 (Mobile Action Technology, Taiwan) and CatLog Gen2 (Catnip Technologies, Hong Kong) GPS loggers. Loggers were sealed in heat shrink tubing to prevent saltwater intrusion and attached to the upperparts of 3–4 central tail feathers using Tesa tape (Tesa Tape Inc., Charlotte, NC). Most GPS devices (42) were programmed to sample every 2 minutes, but a small number (3) collected locations every 30 seconds; all tags lasted for 5 to 10 days. Microbial samples of tagged gulls were acquired from cloacal swabs using sterile cotton swabs (BBL^TM^ CultureSwab^TM^ EZ #220144) and stored on ice until the end of the field day. Swabs were stored in a -20°C freezer within 12 hours of collection and were transferred to a -80°C freezer for long-term storage as soon as possible (maximum of 10 days later for TN samples).

Data collection was conducted according to appropriate national and international guidelines. All sampling procedures and handling of animals were reviewed and approved as a part of the permits obtained. Access to Jamaica Bay Wildlife Refuge and Young’s Island was approved by the National Park Service (Permit: GATE-2017-SCI-0020) and the New York State Department of Environmental Conservation (TRP:2018–001) and relevant bird banding entities (Federal Permit: 22795, DEC Permit: 2036). The sampling protocol for this work was approved by the Stony Brook University Institutional Animal Care and Use Committee (IACUC #: 2016–2247 –Bi -4.19.19-R1)

### Analysis of tracking data

Analyses of foraging trips focused on complete trips (i.e., tracks that originated from and returned to the colony). Since herring gulls occasionally make short movements in close proximity to the colony, only trips in which gulls traveled more than 0.5 km from the colony were considered in analyses.

Locations of foraging behavior along gull tracks were identified as regions of area-restricted search (ARS) using First Passage Time (FPT). FPT is defined as the time required for an animal to move from its current location through a circle with a specified radius. The circle is moved along a path of an animal and FPT is calculated at every point. By doing this at varying radii sizes (10-500m) a scale-dependent measure of foraging activity can be identified [63]. To account for infrequent temporal gaps in GPS data and differences in sampling intervals, points were generated at a 2-minute interval for each track. For each trip, the scale of ARS was identified by locating peaks in the variance of log (FPT) [63,64]. This analysis was conducted for each point along a foraging track, which produced FPT values for overlapping circles. We therefore subsampled the FPT data to reduce spatial autocorrelation as in Suryan et al. (2006) to eliminate points with overlapping circles. For each radius, we first selected the location of maximum FPT and then all other points within a circle that is twice the radial distance from that location were removed. Among the resulting points, we identified points with the maximum FPT values within each circle. The upper quartile of values from the resulting points, representing the most intensive ARS, was then used to identify foraging points and foraging clusters for each trip [64]. Groups of adjacent foraging points identified by FPT analysis that were separated by at least 10 non-foraging points on either end were considered to be foraging clusters. Using this method, multiple foraging clusters were identified for most trips (85%) and foraging clusters were then compared between trips to assess site fidelity to specific spatial locations.

For each foraging trip, foraging points and clusters from the FPT analysis were overlaid on satellite imagery basemaps in ArcGIS software (ESRI World Imagery, May 2017) to identify the overall use of urban environments and to determine the specific habitat type where most ARS points occurred. Urban environments were defined as land-based sites such as shopping centers, parking lots, city streets, landfills, public parks, and other terrestrial suburban or urban areas. We further distinguished terrestrial foraging areas as either landfill habitat, which were identified by locating active public dumps/landfills within the area, or urban habitat, which were defined as any regions of human settlement or development (excluding landfill sites) such as restaurants, parking lots, public parks, and shopping centers. Marsh habitats were identified as aquatic locations of wetlands or coastal areas that were dominated by vegetation or sand, while offshore habitats represented locations in open water that did not occur in a marsh.

To assess whether herring gull foraging behavior varied between colonies, we used linear mixed- effect models (LME) to determine if colony had a significant effect on the following trip metrics: trip duration, trip distance, and maximum trip distance using bird ID as a random effect to account for differences between individual gulls. This approach allowed us to account for the different number of trips taken per bird. We calculated the diversity of foraging habitats used by individual gulls as the degree to which the foraging points identified for an individual occurred within the same habitat type (i.e. landfill, marsh, offshore, urban). To quantify differences in the diversity of foraging habitats used by individuals, we calculated Simpson’s diversity index for each bird using the proportion of foraging points across all trips that occurred in each habitat type. We also assessed the proportion of foraging points that occurred in urban environments for each bird. Lastly, we assessed percent site fidelity for each bird, defined as the proportion of trips that revisited the same spatial location (i.e., foraging clusters that overlapped with one another; if any overlap occurred between clusters, the bird was considered to revisit the same area). We compared foraging diversity values, the mean proportion of foraging points in urban environments, the mean number of trips taken per day, and mean site fidelity between the different colonies with Kruskal-Wallis tests and Dunn’s-tests for post-hoc comparisons using a Bonferroni correction. All analyses were conducted in ArcGIS (v 10.0) and in the R statistical package (v. 3.3.3 R Core Team 2017) using the *adehabitatLT* (v. 0.3.23), *adehabitat* (v. 1.8.20), *rgdal* (v. 1.3–2), *sp* (v. 1.3–1), *chron* (v. 2.3–52), *vegan (*v. 2.5–2), *nlme* (v. 3.1–137), *lubridate* (v. 1.7.4), *MASS* (v. 7.3–50), and *trip* packages (v. 1.5.0).

### DNA isolation, quantification, and sequencing

Microbial DNA was extracted from cloacal swabs for 23 gulls from JB (n = 9), YI (n = 7), and TN (n = 7). We investigated the microbiome of 23 individuals that were selected for analysis due to their differences in foraging behavior within each site. Extractions were performed using a MoBIO Powerlyzer Powersoil Kit (Cat#12855) with several adaptations to the protocol provided by the manufacturer. First, all swab tips were cut off and placed in 1.5mL bead beating tubes with 3mm glass beads and 750μL of bead beating solution. Samples then underwent three rounds of 20 second bead beating cycles and were kept on ice between each round. Following these adapted first steps, the homogenate was transferred to the MoBIO supplied 0.1mm bead tube and incubated with 60μL of the provided C1 solution for 10 minutes at 60°C. After vortexing, centrifugation, and filtration with solution C5 according to manufacturer’s protocol, 75μL of solution C6 was added to the spin filter in a clean 2ml tube and incubated at room temperature for 5 minutes before centrifugation at 10,000g for 30 sec. This extraction process allowed for a final 150μL of solution with DNA from the swabs. Two negative control samples (unused swabs) were run for the DNA extraction kit to account for contaminant DNA associated with the extractions. A QuBit dsDNA HS assay Kit (Life Technologies, Q32854) was used to measure the concentration of DNA in each sample using a QuBit 2.0 quantitation system. DNA concentrations obtained from the swabs ranged from 0.01–16 ng/mL.

The 16S ribosomal RNA gene is highly conserved among bacteria but absent in eukaryotes. Therefore, universal primers have been developed to specifically amplify 16S rRNA genes from widely divergent bacteria and use the resulting sequencing data to extract phylogenetic information and identify the bacteria isolate from any given sample. Bacterial 16S SSU rRNA genes were amplified by PCR using V3 and V4 hypervariable regions with existing primers, (*E*. *coli* 515f and 806r primers as recommended by the Earth Microbiome Project). PCR products were analyzed on a 1% agarose gel stained with ethidium bromide to confirm that extractions contained enough DNA per sample for sequencing and future amplification. Samples were individually barcoded, pooled, and sequenced on an Illumina MiSeq platform (MR DNA Molecular Research, Shallowater, TX) to obtain 250bp paired-end reads.

### Analysis of 16S sequencing data

Analysis and processing of the 16S SSU rRNA gene reads were performed using the open-source QIIME2 pipeline (v. 2017.10) and the *Phyloseq* (v. 1.24.2) package in R. We first used QIIME2 software to demultiplex and quality filter all samples using the DADA2 algorithm. DADA2 applies a quality control process for amplicon data that determines amplicon sequence variants (ASVs) after denoising and filtering out any chimeric sequences and residual PhiX reads, and dereplicating DNA reads. In previous microbiome research, molecular operational taxonomic units (OTUs) used a 97% threshold; however, to avoid the ecological limitations of this method, we applied amplicon sequence variants (ASVs) as recommended in Callahan et al. (2017) [65]. ASVs are a threshold-free metric of classification compared to OTUs. We trimmed the first 50 bases of each read and truncated sequences to 300 bp for DADA2 analysis using the average quality scores for the forward and reverse 250 bp reads (Q-score threshold of 30). We obtained between 47,413 (min) and 88,289 (max) paired-end reads per sample, with an average of 66,188 paired-end reads. ASVs were independently inferred from the forward and reverse of each sample using the run-specific error rates prior to merging read pairs and ASVs were removed if present as chimeric in a sufficient fraction of the samples that they occurred in. ASVs were selected against the Greengenes database (version 13.8 as recommended by the QIIME2 pipeline) using a masked alignment using MAFFT within the DADA2 pipeline and a phylogeny was constructed from the sequences using the QIIME2 FastTree plugin. We then identified and assigned taxonomy to all ASVs using the QIIME2 feature-classifier plugin that uses the Greengenes (version 13.8) database. Lastly, we removed contaminants by identifying any ASVs that occurred in the controls and removed the identical ASVs from the data table of the samples. The resulting ASV table was used for further analysis.

For alpha and beta diversity measurements, each sample was rarified to a depth of 10,000 sequences per sample to avoid biases due to variation in library size across samples. This value was determined using rarefaction curves in order to capture a representative portion of the ASV diversity present by keeping a maximum number of samples in the analysis. *PhyloSeq* was used to calculate core alpha and beta diversity metrics at the ASV level. We estimated alpha diversity using three metrics, Shannon-Weaver (a quantitative measure combining composition and relative abundance) [66], Faith’s Phylogenetic diversity (Faith’s PD; a qualitative measure of biodiversity that incorporates phylogenetic relationships between features) [67], and Pielou’s Evenness (a measure of the evenness of relative abundances). We compared alpha diversity values for all three metrics relative to colony, levels of site fidelity and urban environment use, and predominant foraging habitat type. Post-hoc comparisons were made using the Dunn’s test with a Bonferroni correction when results of Kruskal-Wallis were significant.

The unweighted and weighted UniFrac metrics of microbial community dissimilarity were used to calculate beta diversity and a PERMANOVA was carried out compare beta diversity values between all previously mentioned sample groups. To visualize patterns of microbial dissimilarity in multivariate space, we used principal coordinates analysis (PCoA) and further used paired and unpaired t-tests to assess if axes separated groups of samples using weighted unifrac distances since this metric is a measure of dissimilarity that incorporates phylogenetic distances.

We used *DESeq2* (v. 1.14.1) to investigate differentially abundant bacteria and first modelled counts using a local dispersion model and normalized samples using the geometric mean [68]. Following this, we tested for differentially abundant bacterial taxa between colonies, foraging habitat, levels of site fidelity, and levels of urban environment use; this analysis was run at several different taxonomic levels and grouped accordingly. *DESeq2* uses differential expression statistical Wald tests and corrects for multiple tests using the Benjamini-Hochberg procedure. *DESeq2* also applies a negative-binomial distribution and corrects for outliers. This analysis was completed on unrarefied data. By comparing lists of differentially abundant ASVs, we identified taxonomic richness in samples of each category incorporated in the analysis. Significance was reported as the adjusted p value (p-value with Benjamini-Hochberg correction). The categories used in analyses were colony (n = 9 for JB, n = 7 for YI, n = 7 for TN), foraging destination (the primary habitat type where the highest proportion of ARS points occurred per bird; n = 5 for landfill, n = 5 for marsh, n = 5 for offshore, n = 8 for urban), levels of percent site fidelity (n = 10 for low, n = 8 for medium, n = 5 for high) and the use of urban environments (based on the proportion of foraging points on land; n = 8 for low, n = 10 for medium, n = 5 for high). High, medium, and low categories for site fidelity and urban environment use were defined as 0–33.3% (low), 33.3–66.6% (medium), and 66.6–100% (high) for all metrics.

## Results

### Movement patterns

**Fig 2:**
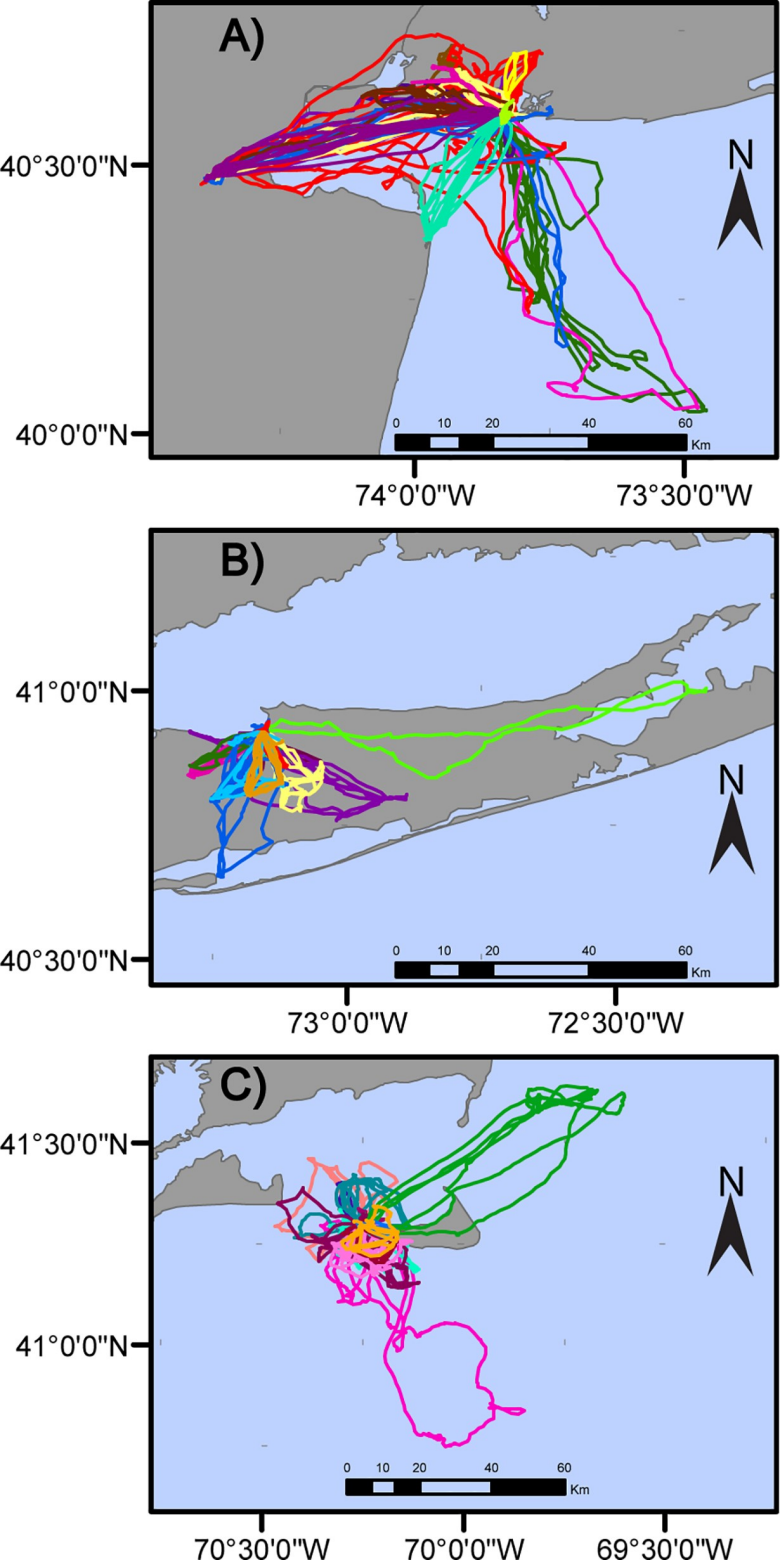
GPS tracks of herring gulls *Larus argentatus* tracked during incubation at three breeding colonies. Tracks were collected from Jamaica Bay (A), Youngs Island (B), and Tuckernuck (C). Each color represents the foraging movements of an individual bird.

**Fig 3:**
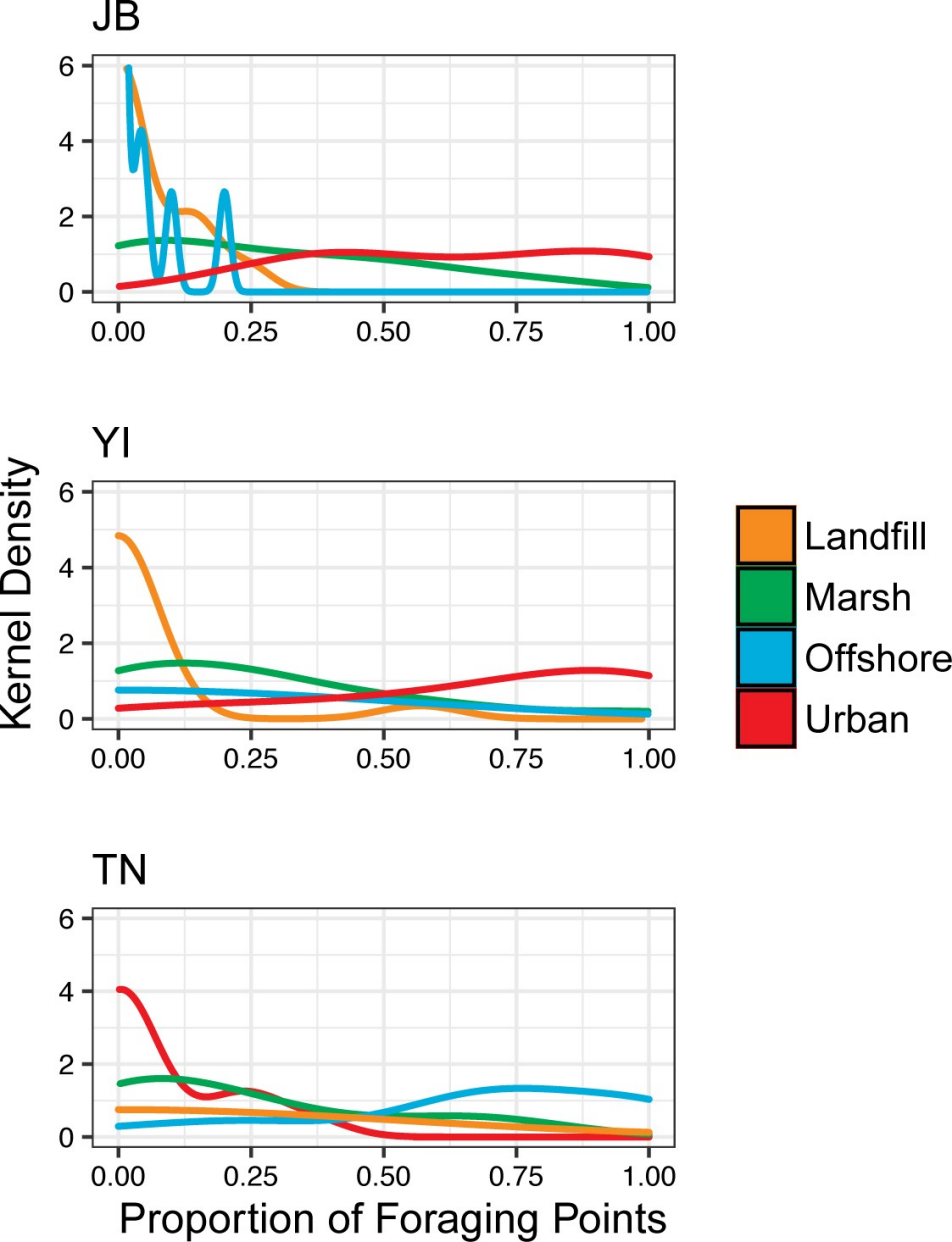
Kernel density plots of the proportion of herring gull foraging points in each habitat type. Habitats used were landfill, marsh, offshore, and urban areas at Jamaica Bay, Youngs Island, and Tuckernuck, respectively.

**Fig 4:**
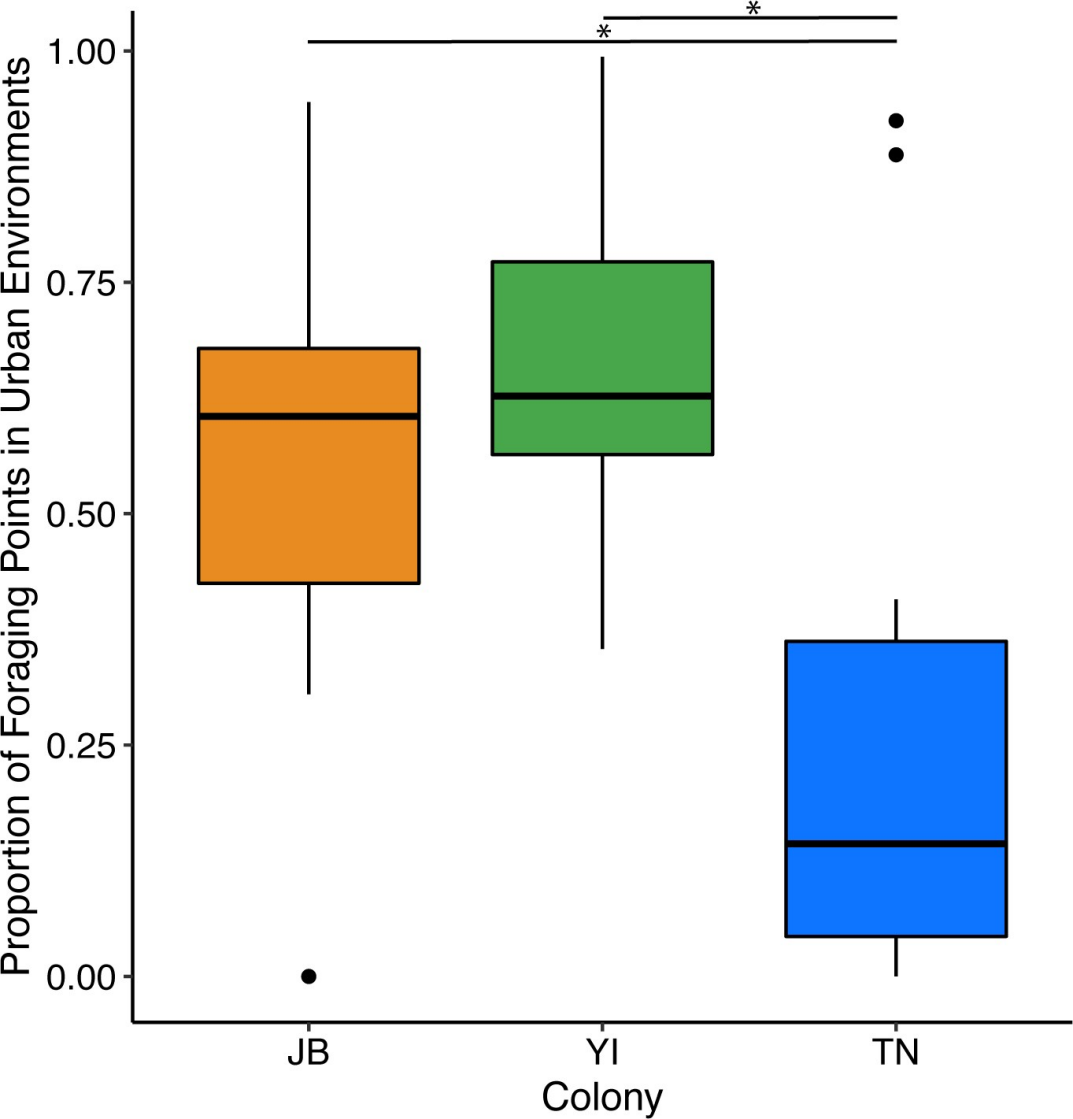
The mean proportion of foraging locations that occur in human habitats at each colony.

GPS tags were deployed on a total of 45 gulls from JB (n = 15), YI (n = 15), and TN (n = 15); (Fig 2), which produced data from a total of 596 foraging trips (JB = 298, YI = 174, TN = 124) (Table A in S1 Dataset). The duration of tag deployments ranged from 4 to 8 days, with 2 tags that lasted for more than 10 days. Tag deployments at JB were slightly longer on average (average duration of 7 days at JB, compared to 5 days for YI and TN), resulting in a larger number of trips observed at that colony. Foraging behavior varied considerably between individuals and colonies. Gulls at all colonies travelled to landfills; the proportion of gulls foraging at landfills during at least one foraging trip was 40% at both JB and TN and 6% at YI (Fig 3). At JB, YI, and TN colonies, 100%, 93%, and 20% of individuals, respectively, used urban habitats to some degree. Gulls at the JB colony foraged primarily in local wetlands, active landfills in northern New Jersey, and urban environments around the NYC area such as public beaches, parking lots, and city streets that often have available discards from restaurants, food stands, and trash receptacles (Fig 2). Gulls from YI only travelled south of the colony and notably did not use the Long Island Sound as foraging habitat, exclusively foraging on land and in nearby marshes around Long Island. The most frequent foraging sites for gulls from the YI colony included coastal salt marshes, suburban shopping centers, and inland parks and lakes. The proportion of foraging areas that occurred in urban environments was high for both JB and YI colonies (Figs 2 and 3). In contrast, TN gulls primarily made long foraging trips to offshore habitats and occasionally used terrestrial sites on Nantucket Island such as a landfill, inland parks, and nearby marshes (Fig 2). There were significant differences in the proportion of foraging points in urban environments between colonies (Kruskal-Wallis p = 0.001, χ^*2*^ = 12.12); YI and JB showed the highest proportion of foraging points in urban environments, while TN showed the lowest; JB (post-hoc Dunn’s test p = 0.02) and YI (post-hoc Dunn’s test p = 0.02) had significantly more foraging points in urban environments than TN (Fig 4).

### Colony-level differences in foraging trip metrics

**Fig 5:**
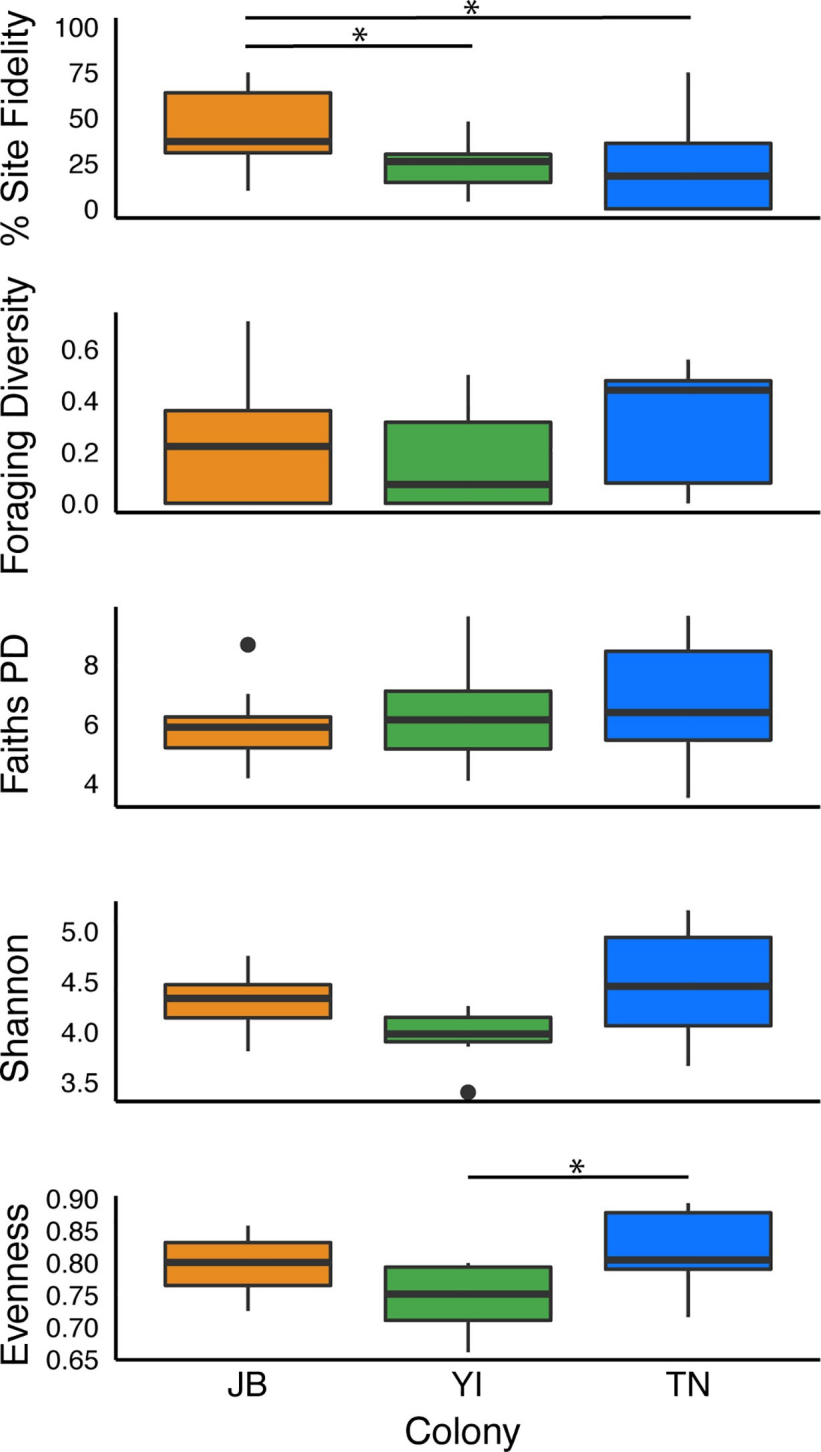
Boxplots of foraging site fidelity, Simpson’s diversity of foraging locations, and microbial alpha diversity metrics. Three metrics of microbial diversity are shown: Faith’s Phylogenetic Diversity (Faiths PD), Shannon’s diversity index, and Evenness (or Palou’s Evenness). Black dots represent outliers in the data and asterisks above the boxes indicate significant differences from Dunn’s post hoc tests using the Bonferroni correction.

When comparing trip metrics at the population-level, colony had a significant effect on total trip duration (LME F_2,40_ = 4.96 p = 0.01) and total distance travelled (LME F_2,40_ = 4.52 p = 0.01). Gull trips from TN were longer in distance and duration; gulls spent 1–2 times more time travelling than JB and YI gulls and for longer distances. There was a weakly significantly relationship between maximum distance traveled and colony (LME F_2,40_ = 2.93 p = 0.06). Mean percent site fidelity also differed significantly between colonies (Kruskal-Wallis p = 0.01, χ^*2*^ = 8.14) (Fig 5). Gulls from JB had significantly higher site fidelity to foraging areas, revisiting 44% of foraging areas on average, than gulls from YI (post-hoc Dunn’s test p = 0.04) and TN (post-hoc Dunn’s test p = 0.01), which revisited 24% and 23% of foraging areas on average, respectively. The diversity of foraging locations did not differ significantly across colonies (Kruskal-Wallis p = 0.12, χ^*2*^ = 9.15) (Fig 5). Lastly, the mean trips taken per day differed significantly between colonies (Kruskal-Wallis p = 0.0008, χ^*2*^ = 14.14; Table A in S1 Dataset). YI gulls took a significantly larger number of foraging trips per day compared to gulls at JB (post-hoc Dunn’s test p = 0.04) and TN (post-hoc Dunn’s test p = 0.02).

### Microbiome composition

**Fig 6:**
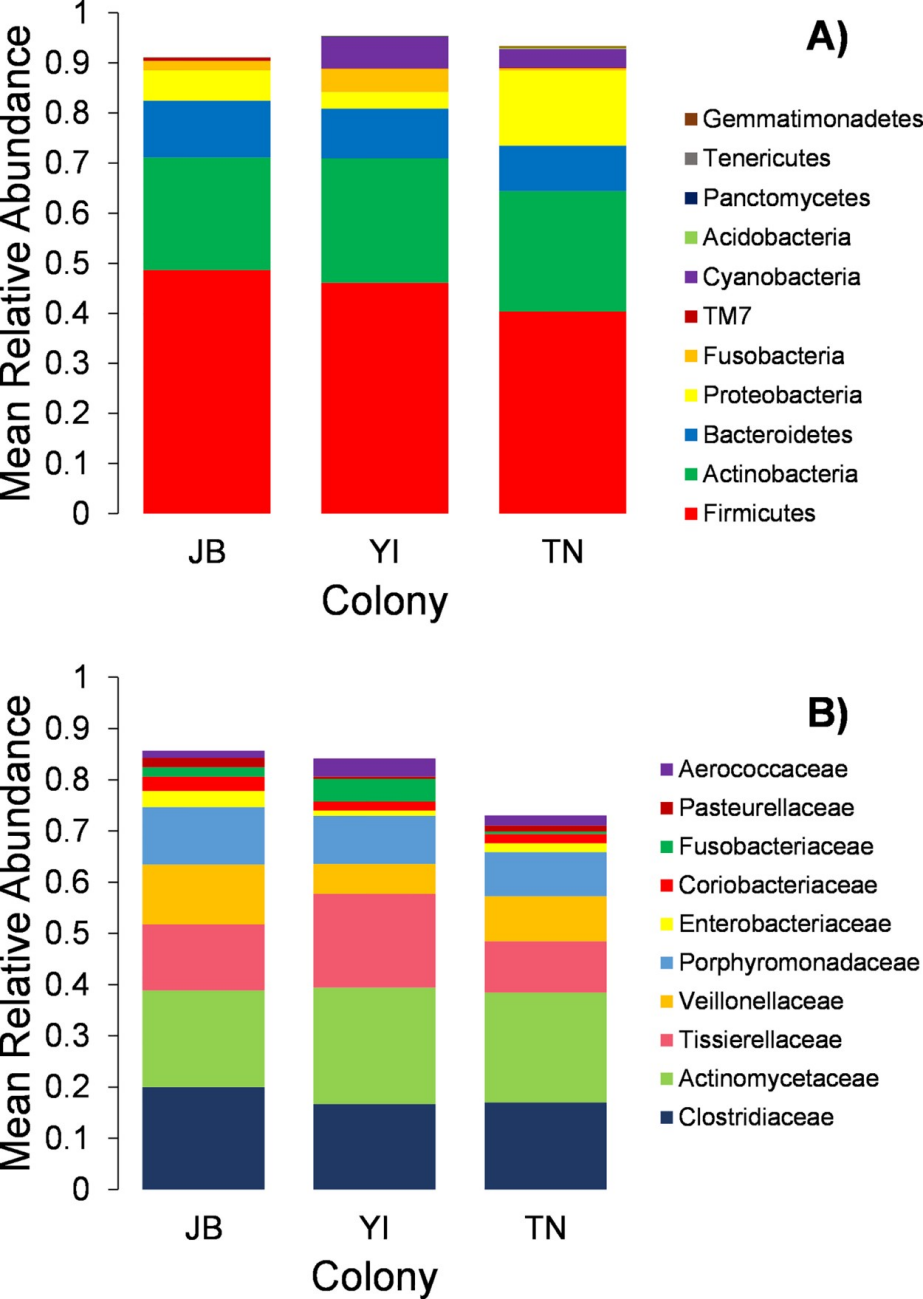
Relative abundances of cloacal bacterial communities by phylum and family. Taxa represented are the most abundant phylum (A) and family (B) for 23 herring gull samples.

Sequencing of 23 of the 45 gulls resulted an average of 66,188 (±SE 18,830) paired-end reads. We detected a total of 1,144 unique ASVs between all samples after removing contaminants (Tables E and F in S1 Dataset). The largest relative abundances in all cloacal communities belonged to the same six phyla, *Firmicutes*, *Actinobacteria*, *Bacteroidetes*, *Proteobacteria*, *Cyanobacteria*, and *Fusobacteria*, which made up over 95% of the cloacal microbiota (Fig 6 and S2 Fig). The bacterial families *Actinomycetaceae*, *Clostridiaceae*, *Tissierellaceae*, *Porphyromondaceae*, *Veillonellaceae*, *Streptophyta*, *Coriobacteriaceae*, *Aerococcaceae*, *Fusobacteriaceae*, and *Enterobacteriaceae* were highly abundant and dominated the highest relative abundances (1–22%) in all samples (Fig 6). *Enterobacteriaceae*, *Campylobacteraceae*, and *Clostridium spp*., families, all known to include clinically important bacteria, were found present in all samples at each colony. More specifically, *Clostridium perfringens*, a bacteria known to act as an avian pathogen in wild birds [69], was found in 45% of all gull samples. *Escherichia coli* [69], a common bacteria that can be transmitted to humans, and the avian pathogen *Campylobacter jejuni* [70–71] were also both present in less than 1% of 80% of samples. It should be noted that these values are minimum estimates of the bacteria present in our samples because many ASVs do not obtain a full taxonomy assignment and only the genus or family names have been assigned.

### Comparative analysis

Initially we compared microbial diversity between sample groups. The overall Shannon diversity index for the microbial cloacal samples was *H’ =* 4.5. Kruskal-Wallis tests comparing alpha diversity across sites were not significant for Shannon (Kruskal-Wallis p = 0.2) or Faith’s PD (Kruskal-Wallis p = 0.1), however, evenness was marginally significant (Kruskal-Wallis p = 0.05), with TN showing significantly higher diversity than YI (post-hoc Dunn’s test p = 0.03) (Fig 5 and Table B in S1 Dataset). Consistently for all three alpha diversity metrics, TN, the least urban colony, exhibited the highest diversity (Fig 5). Principal coordinates analysis (PCoA) conducted on measures of microbial community dissimilarity using weighted unifrac distances showed no clustering of herring gull cloacal samples along any PCoA axes (S1 Fig). T-tests of all principal components also did not exhibit any significant differences between sites or habitat (Table C in S1 Dataset). Furthermore, the PERMANOVA showed that bacterial communities were similarly diverse when using both unweighted and weighted metrics (PERMANOVA; weighted UniFrac p>0.05, unweighted UniFrac p>0.05). PERMDISP tests using both unweighted and weighted UniFrac distances confirmed no significant differences in the dispersion of samples (PERMDISP p = 0.9) (Table D in S1 Dataset).

Next we compared bacterial abundance between sample groups. At the phylum level, only two taxa were differentially abundant between colony and habitat types. *Fusobacteria* was significantly differentially abundant between JB and TN, and between YI and TN. The cumulative relative abundance of this phyla was 1% of JB and 4% of YI samples, whereas in TN, *Fusobacteria* made up only 0.4% (Table G in S1 Dataset). *Fusobacteria* were differentially abundant in urban habitats (4%) in comparison to offshore (0.3%) and landfill (0.3%) samples (Table G in S1 Dataset). *Cyanobacteria* in JB (0.05%) was significantly less than TN (3%) and YI (6%) and was significantly higher in gulls foraging in marsh habitats (9%) than those foraging in urban habitats (0.05%) (Table G in S1 Dataset). Family-level comparisons showed that *Fusobacteriaceae* was differentially abundant between YI (4%) and TN (0.4%) samples (Table H in S1 Dataset). *Oxalobacteraceae* was differentially abundant between several habitat types. In landfill samples, *Oxalobacteraceae* made up less than 0.005% compared to offshore samples where the cumulative relative abundance was 0.2% (Table H in S1 Dataset). *Fusobacterium* was the only differentially abundant variant at the genus-level between colonies exhibiting 0.02% of JB samples and was not present at all in TN (Table I in S1 Dataset). *Amaricoccus* was not differentially abundant between colonies but did show significant differences in abundance between habitat types. *Amaricoccus* was significantly higher in marsh (0.07%) and urban (0.04%) foraging gull samples compared to landfill and offshore foraging gulls where it was not found. It was 50% more abundant in marsh samples compared to urban samples (Table I in S1 Dataset).

## Discussion

Herring gulls have adapted to breed and forage in a multitude of urbanized habitats [4,72]. This study demonstrates that urbanized landscapes impact foraging behaviors of herring gulls, but do not substantially influence the cloacal microbiota. We observed colony-level differences in foraging behaviors of herring gulls; however, herring gulls from all colonies frequently used urban environments, even at the least urban colony. There was no effect of foraging habitat or site fidelity on the microbiome diversity of herring gulls. However, we observed trends in bacterial taxa relative to habitat type and colony, suggesting that habitat use and breeding environment might influence microbiome composition of herring gulls on a fine scale. Interestingly, microbial diversity was consistently highest at TN, the least urbanized colony where individuals used a wider variety of foraging sites and exhibited lower foraging site fidelity. This suggests that urbanization influences foraging effort by providing predictable foraging sites, but that microbial communities may be shaped more by exposure to a variety of spatially discrete foraging sites rather than by habitat type at the scale assessed here.

### Effects of urbanization on herring gull foraging behavior

The degree of urbanization of gull colonies had a significant effect on herring gull foraging behavior. Gulls at the more urban colonies (JB and YI) had considerably higher foraging site fidelity, and took shorter foraging trips than those at the least urban colony (TN). Additionally, gulls from urban colonies primarily foraged at urban environments such as landfills, parking lots, city parks, and shopping centers. At TN, the most remote colony where most gulls foraged at sea, gull trip distances were longer and individuals used a higher diversity of habitats per trip. This suggests that the presence of predictable and consistently available food sources near urban colonies allows herring gulls to develop higher site fidelity and shorten their search effort during foraging trips. Gulls further from urban colonies, such as those at TN, may need to spend more time searching for food due to the lack of reliable fixed food sources. In contrast, the reliance on specific foraging sites was extremely high in JB; gulls at this colony repeatedly foraged at the same foraging areas during successive foraging trips, which is consistent with other seabird studies demonstrating that seabirds display high foraging site fidelity as a response to temporally and spatially consistent food sources [21,73–75]. The high foraging site fidelity observed at JB compared to the other colonies was likely a response to the abundance of long-term stationary food sources around NYC such as a large landfill in northern New Jersey, a popular urban center in Rockaway, NY, and public parks in Brooklyn. Gulls at the YI colony showed the highest proportion of foraging points in urban areas, with no foraging trips going offshore. This pattern was surprising due to the proximity of the YI colony to a large estuary, where birds did not forage, but this behavior may be a reflection of fish and molluscan decline in the Long Island Sound system from eutrophication and overexploitation, which could cause aggregations of foraging gulls to shift towards more dependable urbanized habitats [76–78].

### Herring gull microbial ecology

This study is the first that we’re aware of that uses ASVs, a recently developed and increasingly used technique for bacterial classification, to analyze seabird microbiota. While there are few microbiota studies to date using ASVs since the technique is so new, the DADA2 pipeline is now the preferred method since it classifies bacteria down to the single nucleotide level, allowing for objective, repeatable, and comparable methods for microbial studies unlike the use of OTUs [65]. We found that the herring gull cloacal microbiome is dominated by *Firmicutes*, *Proteobacteria*, *Bacteroidetes*, *Actinobacteria*, *Fusobacteria*, and *Cyanobacteria*. This is similar to the microbiome of other wild avian species [79–81], including other seabirds [82–85]. Interestingly, *Catellicoccus marimammalium*, which is highly abundant in European herring gulls, was not found in any of our samples [58]. *Fusobacteria*, a phyla known to be associated with carnivorous and omnivorous foraging behaviors [41], was more abundant in urban colonies, suggesting that scavenging behaviors in gulls may be more prevalent in urbanized environments. The significantly higher abundance of *Amaricoccus*, a bacteria that has been associated with human waste/sludge [86–88], in landfill and urban habitats may be reflective of the waste present in these locations. With these differences in mind, the use of urban environments by all gulls could explain the overall similarity of bacterial genera composition present if gulls are visiting similar foraging sites outside of the temporal extent of our data. However, the lack of significant differences in beta diversity could also be due to the limited sample size for our microbiome sample groups.

### Effect of foraging environment on gull microbiome

Urbanization may affect overall foraging effort by providing predictable feeding sites, but we suggest that microbial community diversity may be driven by the use of different foraging sites and foraging habitat types, which could lead to an increase in bacterial exposure. Cloacal samples had similar bacterial diversity and composition across colonies and among foraging habitats. Although diversity was significantly different for only one of the three diversity metrics evaluated (evenness), it was consistently highest at TN, the colony with the least urban influence and where individuals used a wider variety of foraging sites with lower foraging site fidelity. This could indicate that increased foraging location diversity and inconsistencies in foraging location selection around TN contribute to a more diverse microbial community compared to JB and YI. The degree of urbanization can substantially affect the microbial communities of wildlife [30,31,89,90]; thus, the overall high microbial diversity in our samples could be explained by foraging in more urban environments to some degree by all tagged gulls. It has been postulated that increased microbial diversity could also be attributed to the contamination or parasitism of an animal [51,91], which could explain the high microbial diversity seen in herring gulls which can acquire parasites, natural infections, and other physiological stressors from urban habitats [92–94]. More research investigating the host-microbiota interactions of gulls would confirm if urban environments, parasites, or pathogenic bacteria cause high microbial diversity in gulls.

For this study, we analyzed the cloacal microbiome specifically because of its role in gastrointestinal processes, which are directly linked to diet. However, there are limited data on the temporal scale of the microbial community in wild birds. Because microbial communities are influenced by short-term changes to diet, physiology, host environment and season [95–97], it is possible that the herring gull microbiome may change between seasons or at shorter temporal scales (e.g., during different breeding stages). Further, gulls are temporally and spatially constrained to forage from the colony in the breeding season, but during the non-breeding season gulls exhibit more variable movements. Thus, herring gulls may exhibit more variable microbiome communities outside of the breeding when they are not constrained spatially. Further research could investigate this with a comparative study of cloacal microbiomes of herring gulls during the spring and winter when gulls are not limited to central place foraging and are not experiencing changes in physiology from breeding requirements.

### Presence of potential pathogens

In the United States, there is a growing concern about the increase of metropolitan birds as vectors for bacteria pathogenic to both humans and other wild animals [15,31,32,98]. The presence of several potentially pathogenic bacteria in our herring gull samples highlights the need to use additional methods to investigate the pathogenic abilities of these bacteria. Metabarcoding does not provide sufficient evidence for bacterial pathogen presence detection. For example, not all *Clostridium* are harmful and phylogenetic analyses could be used to determine if bacterial variants present are related to previously known avian pathogens. Other *Larus* gull species are known to carry pathogenic bacteria [54]; thus, our results reinforce the importance of understanding gulls as vectors for disease transmission. Gulls interact frequently with humans and other birds, often sharing the same physical spaces, such as public recreational beaches and parks [15,99,100]. This coexistence is essential to address when quantifying the effects of gull movement on disease risk for humans and wild animals.

### Other potential factors influencing colony-level differences in foraging behavior

Gulls in larger colonies may experience increased competition, and spatial segregation as a means of avoiding competition may have played a role in colony-level differences in trip parameters since our sites had different gull population sizes [42,72,101]. Although we did not quantify associations with fishing vessels, fishing vessels that occurred south of the JB and TN colonies were likely were destinations for some offshore foraging trips and may provide predictable foraging regions at short time scales. For example, several gulls from TN foraged south of the island near an area frequented by a large commercial squid fishery in late May-late June. The squid boats off TN tend to line up along a 3-mile contour, and some of the gulls foraging south of the island may have been foraging in association with these boats. *Larus* gulls are well-known for taking advantage of fisheries discards [102–104], as they provide abundant marine prey with minimal effort, but future research could explore if gulls from these colonies use available discards throughout the entirety of the breeding season or if there is a shift in diet as chick rearing begins. Foraging on discards may also affect the microbiota if individuals are consuming prey species such as squid or fish that they would not be able to capture otherwise via surface feeding. In addition, *Clostrium* can be present in decomposing carcasses, thus dead fish from discards could have had an impact on the microbiome of herring gulls feeding on them if they were colonized by the bacteria [105]. The sample size of this study was limited, particularly in terms of microbiome samples, and expanding on these findings with an increased sample size could provide more insight into the trends seen in herring gull behavior and microbiome characteristics.

## Conclusions

This study integrated foraging and microbial ecology along an urban gradient and provides a unique example of how spatial and microbiome data can be combined to examine the impacts of urbanization on foraging gulls. Our results suggest that the predictability of food sources in urban habitats influences gull foraging behavior and potentially microbiota; gulls foraging in more diverse habitats showed higher levels of microbial diversity, even if they bred in less urban environments. The high mobility observed in gulls, combined with their high microbial diversity, particularly in our least urban colony, highlights the need to consider foraging movements of animals when studying microbial transfer within and between species. These results will help better understand how reliable food resources may be more profitable for individuals near urban gull colonies. Additionally, our work highlights the potential for parasite and pathogenic interaction within these urban environments. These efforts elucidate the impacts of human development on animal movement and health for species well-adapted to an urban world.

## Supporting information

S1 Fig PCoA analysis of weighted unifrac distances of bacterial community dissimilarity in herring gull cloacal samples. (TIF)

S2 Fig Taxonomy bar plots of individual herring gulls samples at the phylum level. (PNG)

S3 Fig Relative abundances of significantly differentially abundant bacteria at the individual sample level between habitat type and colony. (TIF)

S4 Fig Image of herring gull profile. (JPG)

S1 Dataset Tables A-J include summary data of foraging parameters, alpha diversity, beta diversity, classified bacteria, and differential abundances. (XLSX)

## Abbreviations

FPT: First Passage Time
ARS: Area-restricted search
ASV: Amplicon sequence variant
OTU: Operational Taxonomic Unit
NYC: New York City
JB: Jamaica Bay
YI: Young’s Island
TN: Tuckernuck Island
PCoA: Principal Coordinates Analysis
NMDS: Non-metric multidimensional scaling
ANOSIM: analysis of similarities
PERMDISP: permutational test for homogeneity of multivariate dispersions
PERMANOVA: permutational analysis of variance
