# Supplementary figures and images for: Effects of urbanization on the foraging ecology and microbiota of the generalist seabird Larus argentatus
Source: PLoS One. 2018 Dec 18;13(12):e0209200. doi: 10.1371/journal.pone.0209200 (PMC6298667; doi:10.1371/journal.pone.0209200)

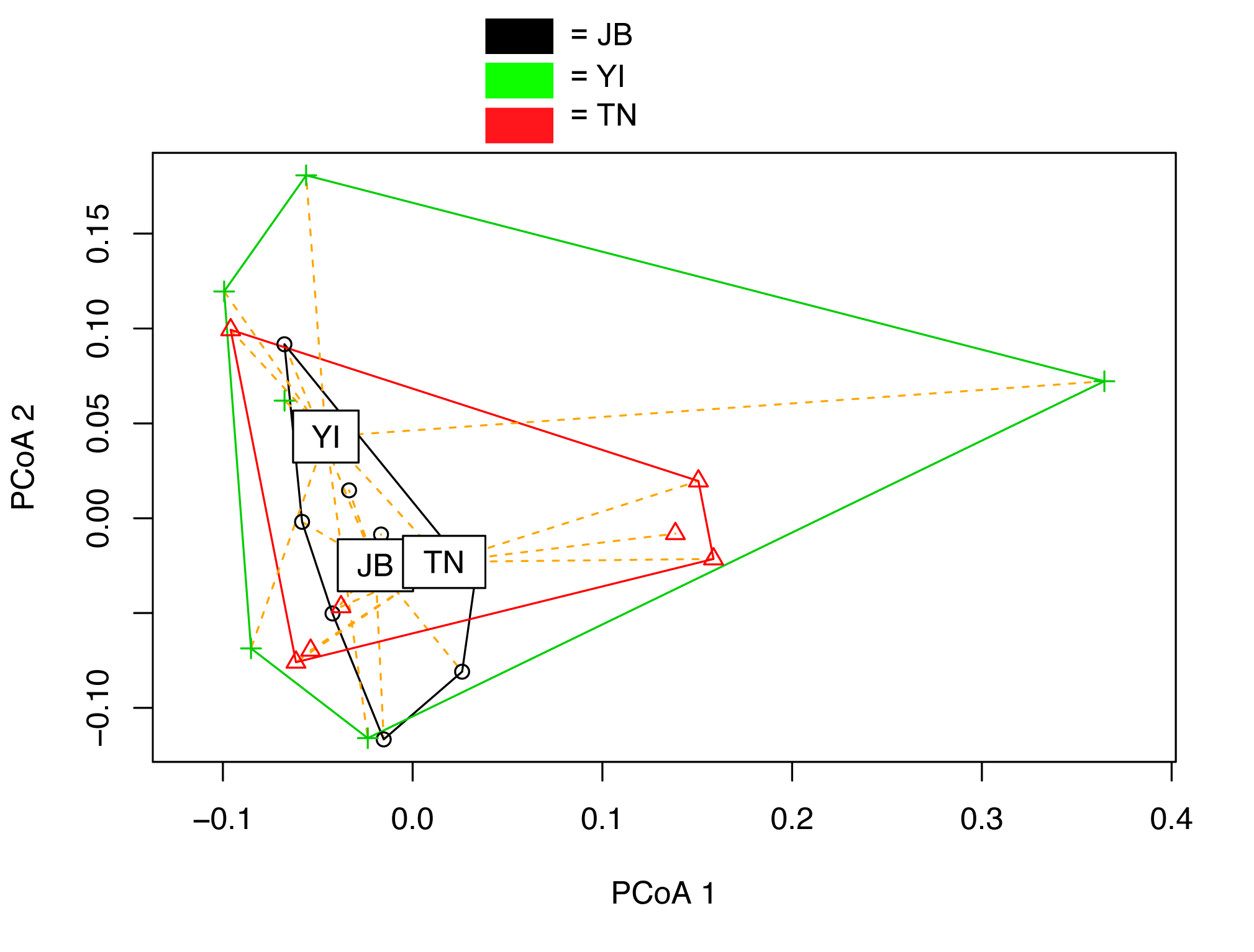

Supplement: S1 Fig — (TIF) [file pone.0209200.s001.tif]

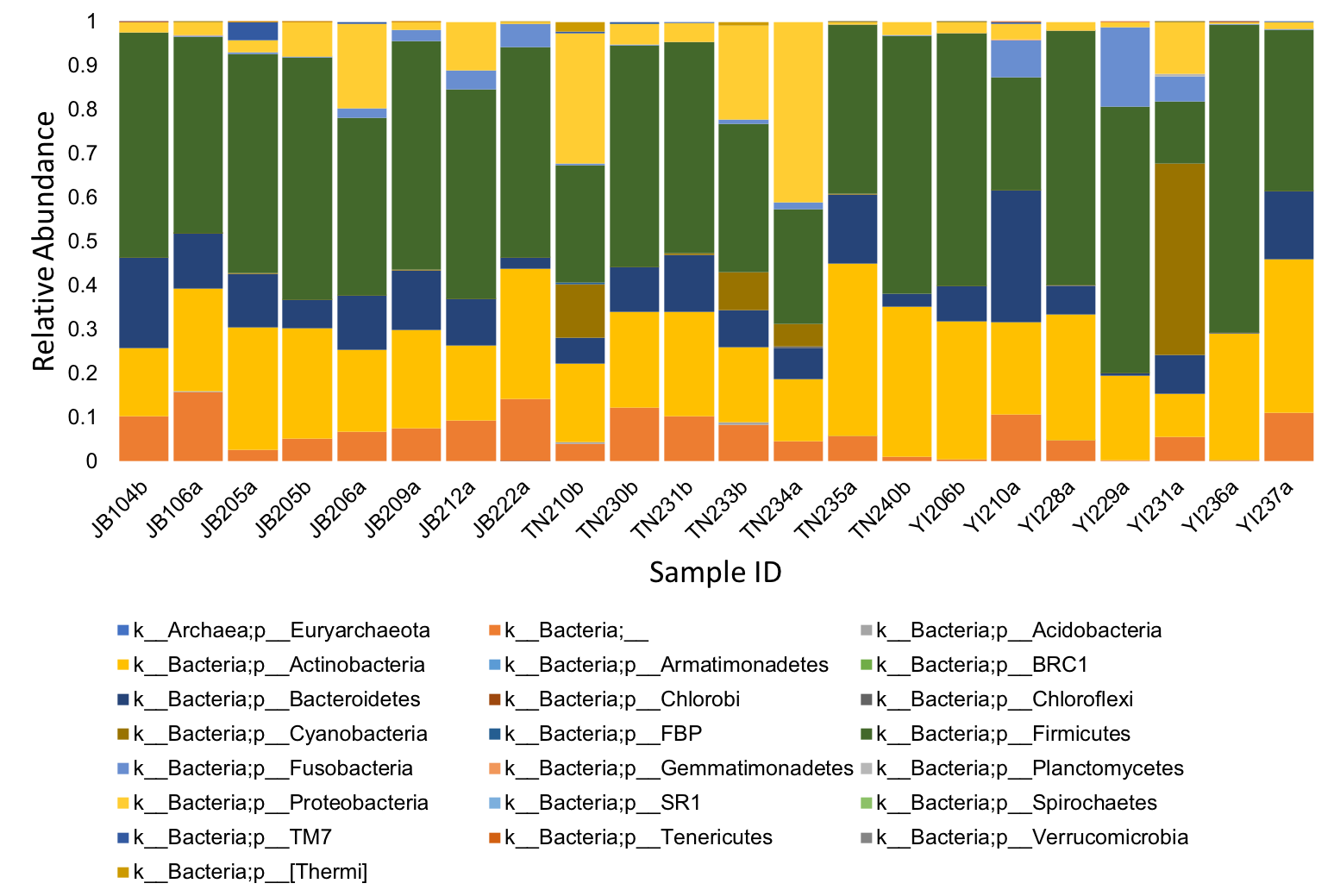

Supplement: S2 Fig — (PNG) [file pone.0209200.s002.png]

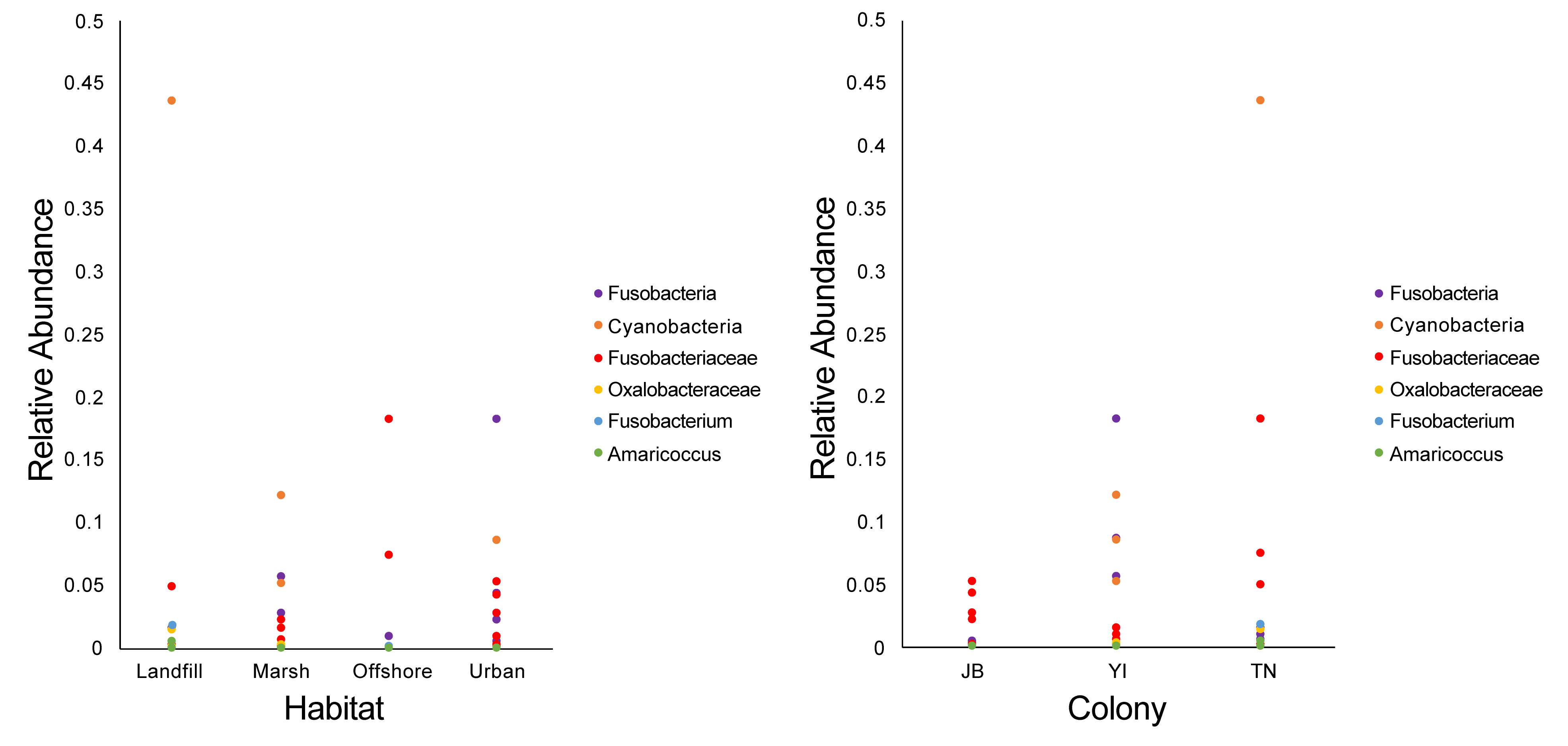

Supplement: S3 Fig — (TIF) [file pone.0209200.s003.tif]

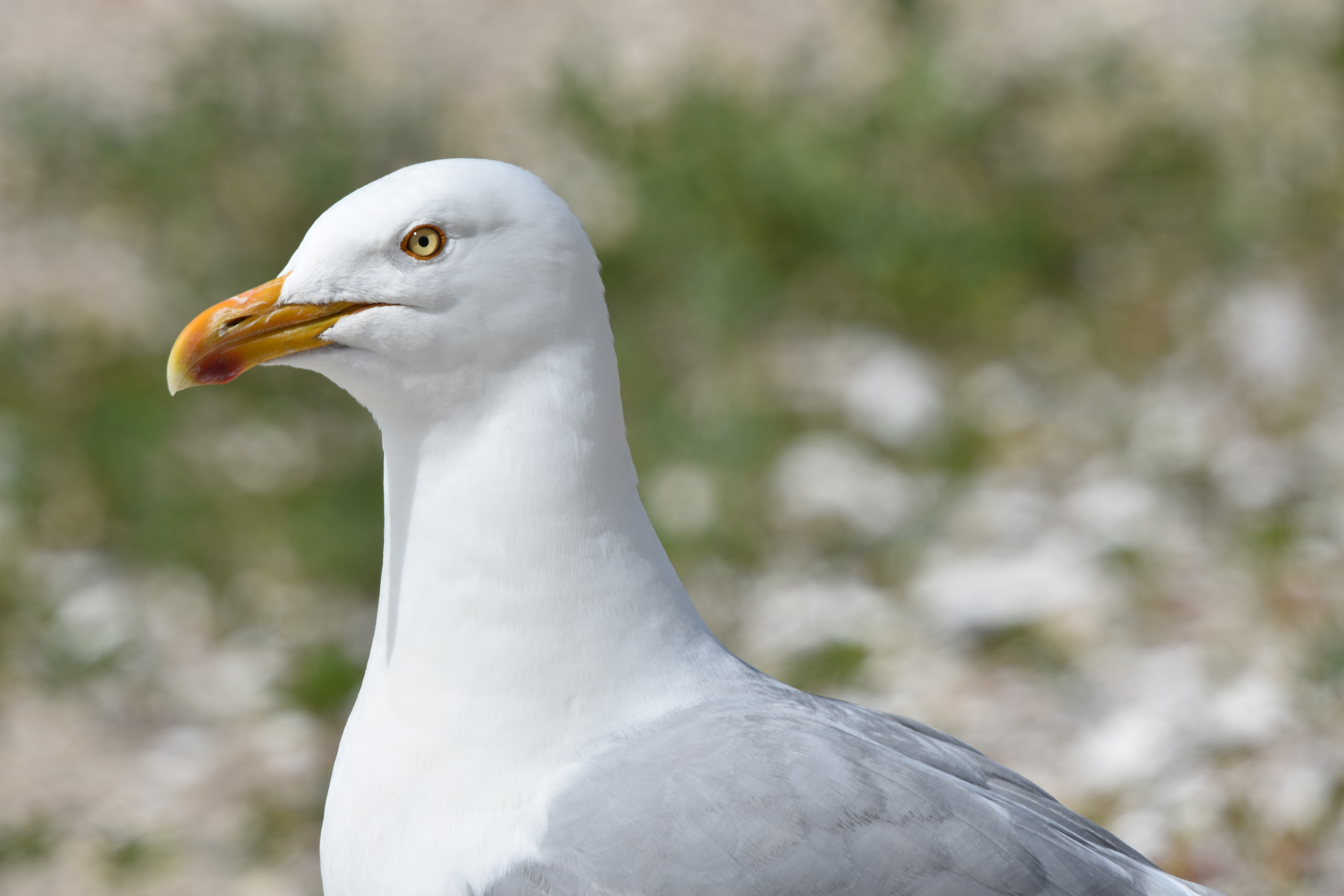

Supplement: S4 Fig — (JPG) [file pone.0209200.s004.JPG]
